# The differential impact of COVID‐19 on the work conditions of women and men academics during the lockdown

**DOI:** 10.1111/gwao.12529

**Published:** 2020-09-03

**Authors:** T. Murat Yildirim, Hande Eslen‐Ziya

**Affiliations:** ^1^ Department of Media and Social Sciences University of Stavanger

**Keywords:** academics, COVID‐19, daily routines, gender, housework, lockdown

## Abstract

That the COVID‐19 pandemic has affected the work conditions of large segments of society is in no doubt. A growing body of journalistic accounts raised the possibility that the lockdown caused by the pandemic has affected women and men in different ways, due mostly to the traditionally gendered division of labour in society. We attempt to test this oft‐cited argument by conducting an original survey with nearly 200 academics. Specifically, we explore the extent to which the effect of the lockdown on childcare, housework and home‐office environment varies across women and men. Our results show that a number of factors are associated with the effect of the lockdown on the work conditions of academics at home, including gender, having children, perceived threat from COVID‐19 and satisfaction with the work environment. We also show that having children disproportionately affects women in terms of the amount of housework during the lockdown.

## 1 INTRODUCTION

That the lockdown caused by the COVID‐19 pandemic has had differential impact on women and men across the globe has received much recognition. The closure of schools and day care facilities has dramatically increased childcare responsibilities, impacting parents’ division of labour at home significantly. Recent accounts have shown that the work and family boundaries became indistinct, and the gendered distribution of responsibilities within the household became more apparent (Alon, Doepke, Olmstead‐Rumsey, & Tertilt, 2020; Cui, Ding, & Zhu, 2020). Some accounts go so far as to suggest that gender inequalities worsened during the lockdown (Minello, 2020). For working women, this typically meant increased responsibilities as the main care provider and as an employee who needs to work from home. Previously described as the double burden or the second shift, this brought forth an overwhelming demand from both family and work (Hochschild & Machung, 2012).

In an attempt to understand the extent to which the pandemic‐related lockdown has affected women and men working in higher education, we designed a survey that asked a series of questions related to the experiences of cademics from various countries, including Norway, Sweden, Italy, France, Germany, the United States and the UK, among others. In particular, we examined the correlates of perceived changes in housework and childcare responsibilities, as well as the work conditions of academics. Our results from a series of ordered logistic regressions indicate that having children is the most important predictor of perceived changes related to work and housework, with women reported being more heavily affected. Perhaps more importantly, we show that the lockdown's impact on individuals varied significantly by whether one had children. Specifically, women with children have reported being affected considerably more, compared with individuals without children.

## 2 THEORETICAL EXPECTATIONS

In this article, we take critical gender theory as the basis of our theoretical framework where gender is defined as a social constructed definition of biological sex where dos and don’ts of masculinity and femininity are shaped by cultural ideals and social institutions (Acker, 1990; Connell, 2002; West & Zimmerman, 1987). Such gendered construction in return results in creating and maintaining structural inequalities at all levels among which higher education institutions are no exception. In fact, universities have long been gendered with strong hierarchy and inequality between women and men academics, with the gap remaining wide in favour of the latter group (O’Hagan et al., 2019). Such structural inequality gets even more enhanced once women have children and caring responsibilities at home. This double burden constitutes one of the obstacles towards the work–life balance where the negative spillover between paid work and domestic duties influences women enormously (Fleetwood, 2007).

We argue that the lockdown caused by the pandemic has worsened this dynamic. For instance, Jessen and Waights (2020) report that working mothers combined childcare and homeschooling with their paid work during this period by working long hours in the evening. Likewise, Andersen, Nielsen, Simone, Lewiss, and Jagsi (2020) show that the pandemic has led women to devote more time to childcare and homeschooling responsibilities, where men remained relatively less affected. The authors go on to argue that the pandemic had a differential impact on the research productivity of women and men. We join this growing body of research in an attempt to advance our understanding of the pandemic‐related changes in the working conditions of women and men in higher education institutions.

## 3 DATA AND METHODS

To advance our understanding of how the COVID‐19 pandemic has affected the work conditions of academics, we fielded an online survey between 10 and 20 June 2020 via a cloud‐based survey platform. In addition to commonly used sociodemographic questions, the survey asked respondents a wide range of questions concerning their perceptions of the work environment at home during the lockdown. We circulated our survey both within and outside our networks, mainly by posting our survey on the social media pages of various academic organizations. In total over 460 respondents have engaged with our survey, where 42 per cent of the respondents (*n* = 198) have completed it. Among those who completed the survey, 65 per cent were women and 55 per cent were social scientists. Slightly more than half of our respondents hold a permanent position, where those with less than five‐year post‐PhD experience constitute around 30 per cent of our sample. Finally, our sample is highly diverse in terms of the country of residence of our respondents; about 90 per cent of our sample consist of academics working in France, Germany, Italy, Norway, Sweden, Turkey, UK and the United States. We report the descriptive statistics of key variables in Table 1.

**TABLE 1:** Descriptive statistics

Variable	Obs	Mean	Std. dev.	Min	Max
Perceived threat from COVID^a^	230	0.296	0.457	0	1
Home‐office^b^	223	3.816	1.169	1 (not at all)	5 (a great deal)
Housework^c^	209	3.522	1.248	1 (not at all)	5 (a great deal)
Childcare^d^	101	3.515	1.411	1 (not at all)	5 (a great deal)
Contribution to chores^e^	205	2.502	0.607	1 (less of my time)	3 (more of my time)
Satisfaction with economic wellbeing^f^	200	3.275	1.098	1 (not at all)	5 (a great deal)
Single income	200	0.355	0.48	0	1
Satisfaction with workspace at home^g^	188	3.202	1.124	1 (not at all)	5 (a great deal)
Tenured	184	0.516	0.501	0	1
Age	184	2.897	1.038	1	5
Without child	253	0.356	0.48	0	1
Full professor	253	0.17	0.376	0	1
Social scientist	253	0.379	0.486	0	1
Gender (1 = woman)	253	0.474	0.5	0	1

^a^ Are you concerned that COVID‐19 is more dangerous for you as an individual?

^b^ How did COVID‐19 influence the time that you are spending on your work?

^c^ How do you think the COVID‐19 pandemic has changed your routines in housework?

^d^ How do you think the COVID‐19 pandemic has changed your routines in childcare?

^e^ Compared to what it was prior to the outbreak, your contribution to house chores is taking: [less of my time, same amount of time, more of my time].

^f^ How satisfied are you with your economic wellbeing?

^g^ How would you rate your satisfaction with your workspace at home?

We utilize four ordinal dependent variables that measure the perceived changes in housework and work conditions after the pandemic. We specifically asked how the pandemic has affected the respondent's (i) time spent on work; (ii) routines in housework; (iii) routines in childcare; as well as (iv) how their contribution to housework has been affected by the lockdown. Accordingly, we estimate a series of ordered logistic regressions, where we control for a number of factors such as family income (single income = 1), satisfaction with home‐office, satisfaction with economic wellbeing, having children, holding a tenured position, age and being a social scientist.^1^

## 4 RESULTS

We report our findings from a series of ordered logistic regressions in Table 2, where we explore how the lockdown has affected academics’ work time (Model 1), routines in housework (Model 2) and in childcare (Model 3), and perceived changes in the contributions to housework (Model 4) during the period they worked from home. As seen in the models, the gender variable is positive in all four models and statistically significant in two of the models, indicating mixed evidence that the pandemic has disproportionately affected the work conditions of women academics during the lockdown, compared with their male counterparts. In particular, women reported being affected at greater rates in terms of their routines in childcare (*p* < 0.01) and in housework (*p* < 0.1). Furthermore, academics without children reported being affected significantly less while those who perceived greater risk from COVID‐19 reported being affected significantly more by the lockdown. Satisfaction with the work environment at home and with economic wellbeing appears to be important factors in explaining perceived changes in one experience with the lockdown, though they come up statistically significant only in two models and at the *p* < 0.1 level.

**TABLE 2:** The impact of COVID‐19 on the daily routines of academics

	Effect on work from home	Routines in housework	Routines in childcare	Contribution to housework
	Model 1	Model 2	Model 3	Model 4
Women	0.515* (0.308)	0.477 (0.293)	1.208*** (0.449)	0.417 (0.330)
Without child	−0.541* (0.302)	−0.836*** (0.298)		−0.838** (0.332)
Single income household	0.602* (0.308)	0.361 (0.295)	−0.448 (0.527)	−0.180 (0.325)
Satisfaction with workspace at home	−0.259* (0.137)	0.0206 (0.126)	−0.347* (0.210)	−0.0162 (0.141)
Satisfaction with economic wellbeing	−0.278* (0.144)	−0.189 (0.135)	0.0555 (0.197)	−0.00661 (0.152)
Perceived risk from COVID‐19	0.566* (0.336)	0.828*** (0.316)	0.877* (0.519)	0.710** (0.359)
Full professor	−0.0870 (0.414)	−0.0459 (0.395)	−0.572 (0.515)	−0.277 (0.439)
Tenured	−0.205 (0.338)	−0.259 (0.330)	0.635 (0.473)	−0.184 (0.370)
Social scientist	−0.0466 (0.282)	−0.0601 (0.274)	0.492 (0.429)	−0.0597 (0.306)
Age	0.00183 (0.175)	−0.202 (0.164)	−0.804*** (0.266)	−0.0213 (0.186)
Constant cut 1	−4.225*** (0.820)	−3.409*** (0.767)	−4.038*** (1.191)	−3.030*** (0.858)
Constant cut 2	−3.651*** (0.798)	−2.321*** (0.745)	−3.280*** (1.162)	−0.556 (0.816)
Constant cut 3	−1.989*** (0.760)	−1.137 (0.731)	−2.432** (1.131)	
Constant cut 4	−0.939 (0.746)	0.0381 (0.722)	−0.977 (1.100)	
Pseudo *R* ^2^	0.043	0.034	0.073	0.038
Observations	180	183	89	184

Standard errors in parentheses.

*** *p* < 0.01;

** *p* < 0.05; *p* < 0.1.

* *p* < 0.1.

**FIGURE 1:**
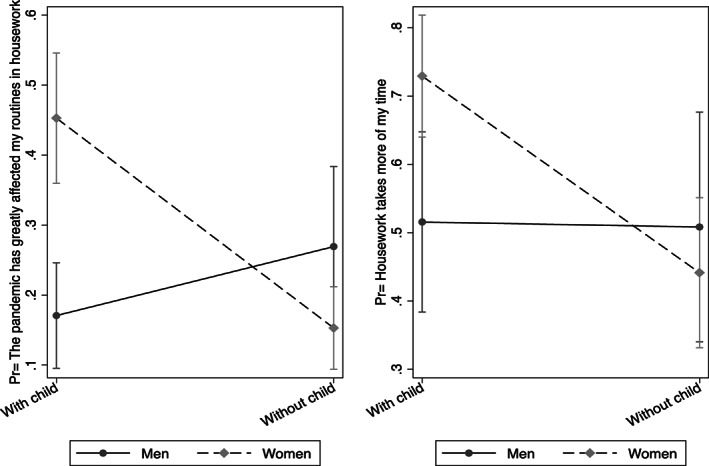
The interactive effects of gender and having children on housework

Table 2 shows that having children appears to be one of the most important predictors of the perceived effect of the pandemic. We delve further into this particular finding by interacting the ‘without child’ variable with gender to explore whether having children affects female and male academics in similar ways. Figure 1 illustrates the substantive impact of gender on daily routines at home across households with and without children, where the predicted outcomes are ‘COVID‐19 has greatly affected my routines in housework’ and ‘my contribution to housework takes more of my time after the pandemic’. As the figure on the left‐hand side shows, women with children have stated at greater rates that the pandemic has affected their routines in housework. In contrast, the gender gap is statistically indistinguishable among households without children. While the figure on the right‐hand side shows the gender gap in the likelihood of saying ‘housework takes more of my time after the pandemic’ among the couples with children almost disappears, it is still clear that having children has no impact on the housework routines of men. What is more, relative to women without children, women with children found housework much more time‐consuming.

## 5 DISCUSSION

Our findings based on an original survey with academics show that while the gender gap in the extent to which the pandemic has affected the working conditions of academics is only weak, the gap becomes alarming among academics with children. Specifically, we show that the daily routines of women academics with children have been disproportionately affected by the pandemic‐related lockdown. These findings are greatly in line with the findings suggesting that:

*academic work — in which career advancement is based on the number and quality of a person's scientific publications, and their ability to obtain funding for research projects — is basically incompatible with tending to children* (Minello, 2020, p. 1)

and that having children leads to reductions in the academic productivity of women, but not men (Lutter & Schröder, 2020, p. 442). Our findings also lend strong support to recent research that found similar gender gaps in the broader population (Collins, Landivar, Ruppanner, & Scarborough, 2020). As schools and childcare facilities were closed during the pandemic, households with children were left with childcare responsibilities including their homeschooling on a daily basis. Our findings imply that the traditionally gendered distribution of labour within the household disproportionately affects men and women working as academics, even among dual‐income families. Although our data do not allow us to tell more about the causal mechanism at work, one possibility is that the lockdown may have forced women working in academia to prioritize care‐taking responsibilities in line with ‘cultural ideals of the good mother’ (Collins, 2020; Sutherland, 2010), bolstering the traditional gender roles at home.

In the absence of concrete projections as to when higher education institutions will return to normal, we proceed with caution in interpreting the implications of our findings for the working conditions of academics in the near future. However, the gender gap in perceived challenges related to increased caregiving demands among academics is not likely to wane soon if the pandemic worsens in the coming months to further aggravate the disruption of routines at work and home. The growing importance of distance learning in the coming semester will surely require many academics from across the globe to reorganize their teaching strategy to go online, which might come at the expense of academics’ research activities. Hence, while it is early to tell the long‐term consequences of this trend for academics’ research activity, the gender gap in perceived disruptions in daily routines may translate into gendered disparities in research productivity. Future research delving further into these possibilities might help us better understand how the pandemic has affected, and will continue to affect, families from across the globe.

## DECLARATION OF CONFLICTING INTERESTS

The authors declared no potential conflicts of interests with respect to the authorship and/or publication of this article.

## ENDNOTE

^1^
There is no multicollinearity among our variables. The correlation between tenured and full professor is 0.48, whereas the correlation between full professor and age is 0.47.
